# Developing a prediction model of neonatal pneumonia in patients with gestational diabetes mellitus in primary medical institutions

**DOI:** 10.3389/fped.2026.1840176

**Published:** 2026-08-25

**Authors:** Lian Wu, Qun Cao, Jing Wu, Ting Liao, Li Li, Chengying Yang

**Affiliations:** Department of Pediatrics and Obstetrics, Anyue Maternal and Child Health Hospital, Ziyang, Sichuan, China

**Keywords:** gestational diabetes mellitus, hyperemesis during pregnancy, neonatal pneumonia, prediction model, preterm

## Abstract

**Background:**

The incidence of gestational diabetes mellitus (GDM) continues to rise in China, and the risk of neonatal pneumonia is significantly increased, but the primary medical institutions lack simple and effective early risk assessment methods.

**Objective:**

To construct a prediction model for neonatal pneumonia suitable for primary medical institutions, based on readily available clinical characteristics of pregnant women with GDM, we aimed to identify key risk factors and provide a basis for developing clinical intervention strategies.

**Methods:**

Data on pregnant women and their newborns at Anyue Maternal and Child Health Hospital from 2023 to 2025 were retrospectively collected. Eight machine learning methods were used to construct the prediction model by bootstrap method and SHapley Additive exPlanations (SHAP) was used to enhance the model's interpretability.

**Results:**

A total of 328 pairs of mothers and newborns were included, and the incidence of neonatal pneumonia was 8.8%. Among the eight prediction models, Random Forest model performed the best and Decision tree model was the worst; SHAP results indicated education level, BMI, hyperemesis gravidarum, primipara, hyperlipidemia during pregnancy, premature birth and job were most influencing factors. Binary logistic regression analysis showed that premature birth (OR = 4.763, 95% CI = 1.522−14.901, *P* = 0.007), Hyperemesis Gravidarum (OR = 3.706, 95% CI = 1.587−8.653, *P* = 0.002), and cesarean section (OR = 2.456, 95% CI = 1.050−5.745, *P* = 0.038) were risk factor of neonatal pneumonia, with a protective factor of higher education level (OR = 0.370, 95% CI = 0.158–0.865, *P* = 0.022).

**Conclusion:**

Despite the limited number of neonatal pneumonia events, our findings provide preliminary evidence that it may be feasible to develop a simplified risk-stratification tool for neonatal pneumonia in pregnancies complicated by GDM using routinely available clinical variables. Further multicentre prospective studies are required to determine the optimal predictor set and externally validate the model before clinical application.

## 1 Introduction

Gestational diabetes mellitus (GDM) is a disease caused by maternal insulin dysfunction, manifested as glucose intolerance, that occurs during pregnancy or is first recognised during pregnancy (1). GDM has been associated with a number of adverse outcomes, including preeclampsia, premature delivery, polyhydramnios, and fetal macrosomia in the short term, as well as an increased risk of postpartum T2DM and cardiovascular disease in the long term (2, 3). Furthermore, GDM may lead to an elevated long-term risk of obesity, metabolic syndrome, cardiovascular dysfunction, and neurodevelopmental disorders in offspring (4, 5). Current research indicates that GDM affects approximately 14% of pregnant women worldwide (6), although the diagnostic rate remains below 70%. The incidence of GDM in China has continued to increase in recent years, especially in rural areas (0.90% per year), faster than in urban areas (0.60% per year) (2). The extant literature suggests that GDM may pose a significant threat to maternal and infant health, constituting a pressing clinical concern.

Neonatal pneumonia (NP) is an infectious inflammation of the lung parenchyma or stroma in newborns and is a major cause of death in this age group worldwide (7). The clinical symptoms of neonatal infectious pneumonia are non-specific and are usually manifested as apnoea, shortness of breath, respiratory distress, tachycardia or bradycardia, hypothermia, etc. The early onset of the condition is often similar to the clinical manifestations of neonatal sepsis, and the severe cases can progress to septic shock and acute respiratory distress syndrome (8). The immature immune system of newborns renders them susceptible to bacterial infection, which can result in congenital pneumonia and early-onset pneumonia (9). For instance, the global neonatal infection rate for *Klebsiella* pneumoniae is approximately 0.3% [95% confidence interval (CI) = 0.2%–0.3%], yet it is associated with a mortality rate of around 22.9% (95% CI = 13.0%–32.9%) (10). Furthermore, a multitude of factors, including maternal perinatal factors, neonatal birth weight, seasonal variations, malnutrition, and others, have been shown to increase the risk of neonatal pneumonia (11, 12). The extant literature suggests an urgent need to develop a reliable, clinically accurate diagnostic strategy for neonatal pneumonia to reduce the risk of misdiagnosis and missed diagnoses.

Although several maternal and neonatal predictors of NP have been identified in general birth populations (13), women with GDM constitute a clinically distinct subgroup characterized by chronic maternal hyperglycemia, altered maternal-fetal immune interactions (14), and increased neonatal susceptibility to infection. For example, the maternal hyperglycemic environment can specifically impair fetal immune development via sustained activation of the HIF-1*α* pathway and reduced diversity of the IgG antibody spectrum in cord blood (14), rendering these newborns uniquely susceptible to infection. Consequently, predictors identified from unselected birth cohorts may not accurately reflect the relative importance of clinical risk factors among pregnancies complicated by GDM.

The ongoing proliferation of GDM at the primary level (2) and the inadequate capacity for neonatal infection prevention and management in primary healthcare institutions in China (15) have heightened the risk of neonatal pneumonia. Therefore, this study aimed to develop a prediction model specifically for neonates born to mothers with GDM using eight machine learning algorithms, based on routinely collected clinical variables available in primary healthcare settings. We sought to identify key risk factors that could inform targeted postnatal surveillance strategies and serve as a foundation for future multicenter validation studies.

## 2 Materials and methods

### 2.1 Participants

**Figure 1:**
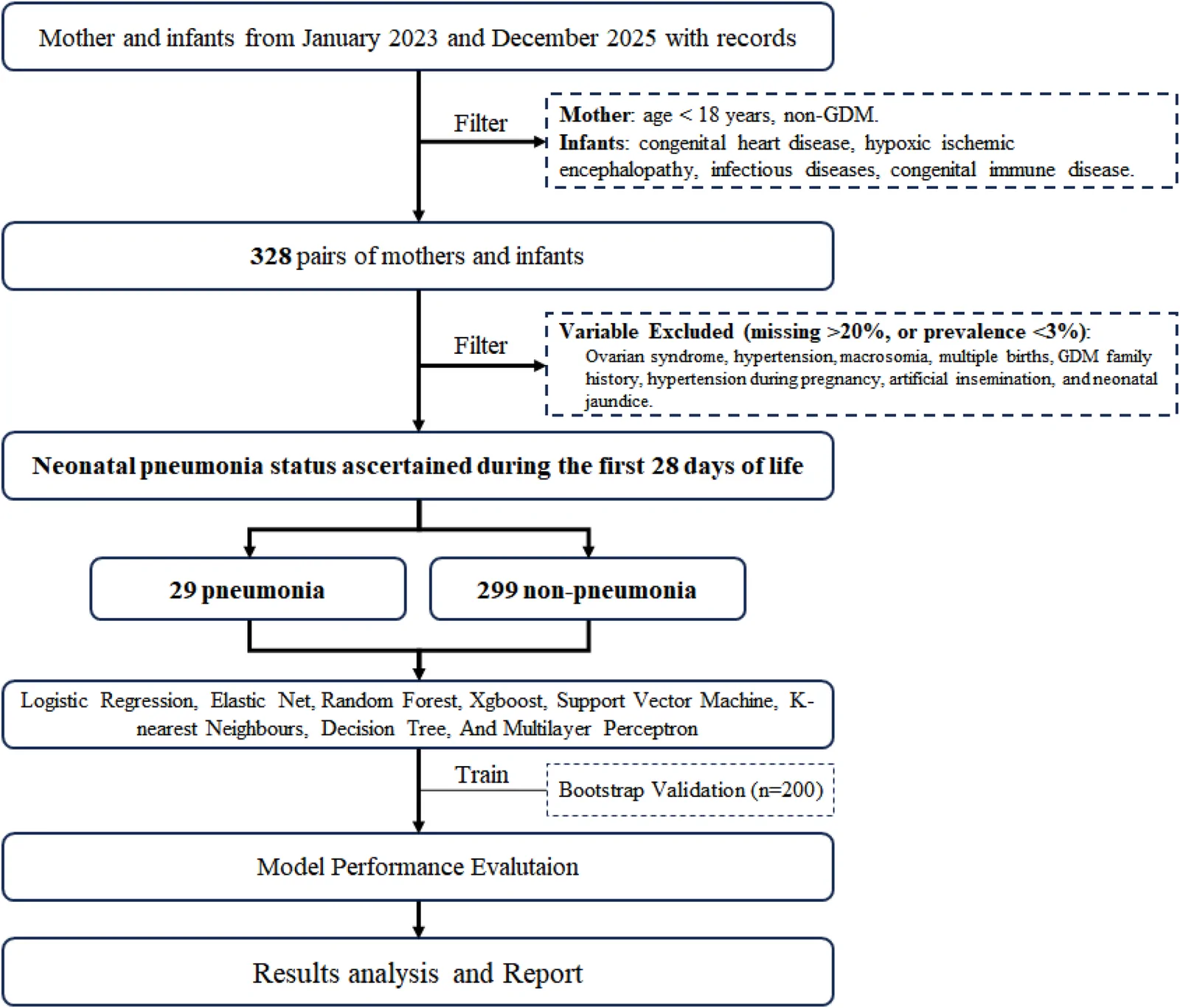
Schematic representation of the workflow of the neonatal pneumonia.

This study was conducted at Anyue Maternal and Child Health Hospital, a county-level primary healthcare institution in Sichuan Province, China, serving a predominantly county population with approximately 1,600 deliveries recent years. As a primary maternal and child health facility, it provides routine antenatal care, delivery services, and neonatal basic care, but lacks advanced neonatal intensive care capabilities—a setting representative of the resource-limited environments where this risk score is intended to be applied. Data on pregnant women with GDM who delivered at this hospital between January 2023 and December 2025 were retrospectively collected. The protocol was in accordance with the stipulations set out in the Declaration of Helsinki and its subsequent amendments. The study was reviewed and approved by the Ethics Committee of Anyue Maternal and Child Health Hospital (approval number: 2026004). The study utilised de-identified historical medical record data exclusively, and it posed no additional risks to patients. Therefore, the Committee had approved the exemption of the informed consent requirement. The main procedure of this work was shown in Figure 1.

**Inclusion criteria** for mother–infant pairs were as follows: (1) maternal age >18 years; (2) the mother met the diagnostic criteria for GDM; (3) neonatal clinical records were available for ascertainment of neonatal pneumonia status during the first 28 days of life (16); and (4) the required clinical data were complete; Neonates who met the diagnostic criteria for neonatal pneumonia described in Section 2.2 within the first 28 days of life were classified as the NP group, whereas those who did not meet these criteria during the same ascertainment period were classified as the non-NP group. **Exclusion criteria** were congenital heart disease diagnosed at birth; hypoxic–ischemic encephalopathy; other infectious diseases such as encephalitis; immunodeficiency; or congenital immune disorders.

### 2.2 Neonatal pneumonia diagnosis method

The diagnosis of neonatal pneumonia was rigorously established according to the 2025 *Guideline for the diagnosis and treatment of common neonatal diseases in primary healthcare institutions: neonatal infectious pneumonia* (8). A confirmed diagnosis required that an infant met all three of the following criteria: 1) documented maternal or perinatal infectious exposure, including maternal fever, chorioamnionitis, prolonged rupture of membranes (>18 h), or microbiologically confirmed maternal infection; 2) Compatible clinical manifestations, including at least one of the following: abnormal breathing (tachypnea, grunting, or apnea), fever or hypothermia, poor response, and auscultatory findings such as thick breath sounds or wet rales in both lungs; 3) Confirmatory chest ultrasound findings, such as increased or blurred markings, patchy shadows, emphysema, or atelectasis (17).

### 2.3 GDM diagnosis

According to the International Association of Diabetes and Pregnancy Research Group (IADPRG) standards (18, 19), a 75-g oral glucose tolerance test (OGTT) was performed at 24 and 28 weeks of gestation. If any time period condition (FPG >= 5.1 mmol/L, one-hour PG > 10.0 mmol/L, or two-hour PG > 8.5 mmol/L) was met, the GDM was confirmed.

### 2.4 Clinical data collection

Clinical data was systematically extracted from electronic medical records and categorized into four domains: Maternal demographics and health history: Age, nationality, pre-pregnancy body mass index (BMI), education (Edu) level, personal disease history (diabetes, hypertension or immune or autoimmune diseases), family history, lifestyle factors (smoking, alcohol consumption). Obstetric history: Primipara (Pri), history of premature birth, history of macrosomia delivery, history of adverse pregnancy outcome (APO) and childbirth. Current pregnancy status: mode of conception, number of fetuses, presence of hyperemesis gravidarum (HD), pregnancy induced hypertension, pregnancy hyperlipidemia (PH), mode of delivery. Neonatal outcomes: Gender, birth weight, premature birth (PB), 1-minute and 5-minute Apgar score, and the occurrence of pneumonia, jaundice or hypoglycemia. Preterm birth was defined as delivery before 37 completed weeks of gestation (<37 + 0 weeks, or <259 days), in accordance with the ICD-11 definition (16).

### 2.5 Prediction model construction

Following the *Transparent Reporting of a multivariable prediction model for Individual Prognosis Or Diagnosis* (TRIPOD) (20) and the recommended construction method for a clinical prediction model (21), the pneumonia model for GDM newborns was gradually established.

#### 2.5.1 Data preprocessing

Variables with a missingness rate greater than 20% will be excluded, and Multiple imputation (MI) was utilised to process the residual missing values. The interpolation method employed was that of predictive mean matching (PMM). Five complete data sets were generated with 20 iterations to ensure the convergence of interpolation. Definition of categorical variables: Edu was dichotomized as Lower (≤9 years, i.e., junior high school or below) vs. Higher (≥10 years, i.e., high school or above). HG was defined by clinical diagnosis requiring ≥3 episodes of vomiting per day with either ≥5% weight loss from pre-pregnancy weight or ketonuria. PH was defined as total cholesterol  ≥ 5.2 mmol/L or triglycerides  ≥ 1.7 mmol/L during pregnancy. Because gestational age is intrinsically a continuous variable, we additionally performed a sensitivity analysis in which gestational age at delivery (in weeks) was entered as a continuous predictor in place of the dichotomous preterm-birth variable. Preterm birth and continuous gestational age were not included simultaneously because the former is directly derived from the latter. The dichotomous preterm-birth variable was retained in the primary simplified risk-score analysis because the <37-week threshold represents an internationally standardized and clinically readily interpretable definition of prematurity (16). Predictor variables with a prevalence <3% were excluded a prior to feature selection because extremely sparse variables may lead to unstable parameter estimation and model non-convergence in small datasets (22). Importantly, this procedure did not result in exclusion of any mother–infant dyads; all eligible participants remained in the analytical cohort unless they met the predefined inclusion and exclusion criteria (23). To assess the validity of the imputation procedure, we compared the distribution of all candidate variables between the original raw dataset and the first imputed dataset (Supplementary Table S1), confirming that the imputation did not significantly alter the data structure.

#### 2.5.2 Feature selection

To identify the predictors most strongly associated with neonatal pneumonia and thereby reducing model complexity, the least absolute shrinkage and selection operator (LASSO) regression was employed for feature selection. The optimal penalty parameter (*λ*) was determined using 10-fold cross-validation to minimise cross entropy. The feature with non-zero retention coefficient was used as the candidate prediction variable of the data set. In the absence of a selected feature in a given data set, the first five variables with the maximum variance were selected as alternatives. Finally, the feature selection frequency was summarised across five interpolated datasets.

#### 2.5.3 The candidate models

The present study employed eight machine learning algorithms, including logistic regression (LR), ElasticNet (EN), random forest (RF), XGBoost (XGB), support vector machine (SVM), k-nearest neighbours (KNN), decision tree (DT), and multilayer perceptron (MLP). LR employed an L2 penalty (ridge regression) and automatically selected *λ* through 5-fold cross validation to prevent complete separation (24). EN employed a composite L1/L2 penalty, with the penalty parameter *α* fixed at 0.5 and the regularisation parameter *λ* selected by cross validation. The number of trees in RF was adapted according to the sample size, with a minimum node size of 1/100 of the sample size (25). XGB algorithm utilised a learning rate of 0.05, a subsampling ratio of 0.7, and a column sampling of 0.7. The optimal number of iterations was determined through early stopping and cross-validation (26). SVM employed a radial basis kernel function, and the kernel parameter *σ* was estimated based on the median distance, with the probability model output. KNN algorithm was employed to select the optimal value of k through a process of simple cross validation (3-fold). DT employed an adaptive complexity parameter approach, with a minimum split sample size of n/50. MLP maximum number of iterations was adjusted based on the number of features, and three different initial values were attempted to select the optimal model (27). Prior to the training phase, all models were standardised on continuous features (i.e., features that take a numerical value) through a process of mean subtraction and standard deviation division. This procedure ensured the comparability of features with different dimensions.

#### 2.5.4 Model training and internal validation: bootstrap optimistic correction

In order to circumvent the issues of overfitting and the partial utilisation of limited samples, the Bootstrap Optimism Correction method was employed to assess the efficacy of the model (28). The following steps must be taken for each interpolated dataset (*n* = 328): 1) Apparent Performance: The model was then trained on the complete dataset, with the prediction probability being calculated. The optimal threshold was then determined based on Youden's J index. 2) The calculation of sensitivity, specificity, accuracy, F1 score, and AUC (Area Under the ROC Curve) was also recommended. 3) The bootstrap resampling method was employed. This process was to be repeated 200 times, resulting in 200 samples of the same size being extracted from the original data with replacement. 4) The model was retrained on each Bootstrap sample. AUC of the model on the Bootstrap sample was then calculated. 5) The model was then used to predict the AUC of interpolation set data. The optimistic value was defined as the difference between two AUCs, and the optimistic correction was calculated as the mean of 200 optimistic values.

#### 2.5.5 Cross interpolation performance merging

For each model, a set of calibrated performance metrics was generated for each interpolated dataset (*n* = 328). The utilisation of the Rubin rule in conjunction with multiple imputations was a subject of considerable uncertainty (29). AUC was the Fisher Z-transform, which was then followed by the calculation of the z-values of each imputation. The mean, intra-imputation variance, and inter-imputation variance were then calculated. The merged z-values were then inverse transformed to obtain the merged AUC and its 95% confidence interval. Proportional indicators (sensitivity, specificity, F1, accuracy, and the Jordan index) were initially subjected to logit transformation, subsequently, the mean of each interpolation transformation value was calculated, and an inverse transformation was performed to obtain the merged point estimate. The calculation of the confidence interval was based on the interpolation variance and t-distribution (with a degree of freedom of m-1). In conclusion, the merged AUC (including 95% CI), sensitivity, specificity, F1 score, accuracy, Youden index, and optimal decision threshold were obtained for each model.

#### 2.5.6 Final model and explanatory analysis

The optimal model was to be determined on the basis of the highest merged AUC. SHAP (Shapley Additive exPlanations) was a game-theoretic approach that assigns each feature an importance value for a particular prediction (15), thereby enhancing model interpretability by revealing the direction and magnitude of each feature's contribution to the predicted outcome.

### 2.6 Statistical analysis

All data analysis in this study was conducted using R 4.5.2, and data visualization and graphical output were performed using RStudio 2026.01.0 (Posit Software, PBC, 250 Northern Ave, Suite 420, Boston, MA 02210). Data organization and preprocessing were performed using tidyverse 2.0.0, missing values were processed using mice 3.17.0 package for multiple imputation, and correlation analysis was performed using corrplot 0.95 package to calculate the Spearman correlation coefficient. Feature selection was performed using the glmnet 4.1.8 package for LASSO regression, sample partitioning was implemented using caret 7.0.1 for stratified sampling. The model construction was based on the tidymodels framework, and each model engine was used separately: glmnet 4.1.8, ranger 0.18.0, XGBoost 1.8.0, kernlab 0.9.33, kknn 1.5.0, rpart 4.1.26, and the Annet package 7.3.20. In the model performance evaluation, AUC and its confidence interval were calculated using the pROC 1.19.0.1 package, and decision curve analysis (DCA) was performed using the dcurves 1.6 package. The interpretability analysis of the model was conducted using the shapviz 0.9.0 package, and the combination of graphics was completed using the Patchwork 1.3.0 package. Contineous variables analyzed by *t*-test, and category variables by *χ*^2^ test. Multivariate analysis was conducted using binary logistic regression with stepwise method. *P* < 0.05 was considered statistically significant.

## 3 Results

### 3.1 Basic information about the participants

**Figure 2:**
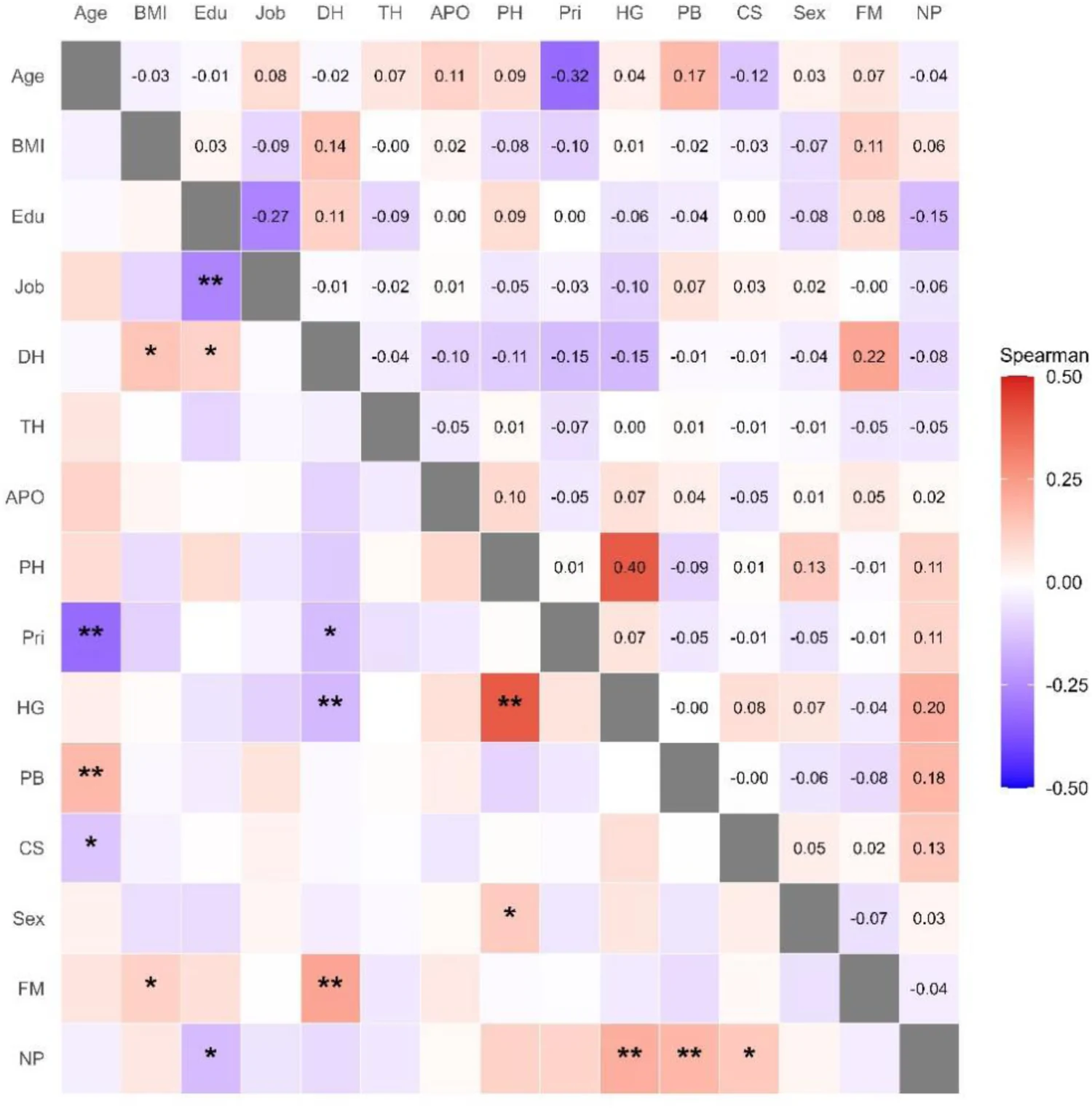
Spearman correlation results of all variables. APO, adverse pregnancy outcome; CS, caesarean section; DH, diabetes history; FM, fetal macrosomia; HG, hyperemesis gravidarum; NP, neonatal pneumonia; PB, preterm birth; PH, pregnancy hyperlipidemia; Pri, primipara; TH, thyroid abnormalities history. * indicates *P* < 0.05 and ** indicates *P* < 0.01.

Twenty-nine cases of neonatal pneumonia were collected from 328 (8.8%) pairs of mothers and children. Several candidate predictor variables with a prevalence below 3% (including ovarian syndrome, chronic hypertension, macrosomia, multiple pregnancy, family history of GDM, pregnancy-induced hypertension, artificial insemination, and neonatal jaundice) were excluded from model development because of sparse data. No mother–infant dyads were excluded on the basis of these rare characteristics. Supplementary Table S1 presented no significant differences were observed between the raw and imputed data for any variable (all *P* > 0.8), confirming that the imputation procedure did not introduce substantial bias and that all 328 mother–infant pairs were retained in subsequent analyses. Table 1 showed there were significant differences in Edu, HG, PB and CS between NP and No-NP groups. As demonstrated in Figure 2, neonatal pneumonia correlated with Edu, HG, PB and CS.

**Table 1:** Basic characteristics of study participants stratified by neonatal pneumonia status.

Variables		Non-NP	NP	T, X2	*P*
Age		31.23 ± 4.75	31.03 ± 6.57	0.160	0.874
BMI		24.31 ± 3.68	25.75 ± 4.72	−1.604	0.119
Education level	Low	94 (85.5%)	16 (14.5%)	6.468	0.011
	High	202 (94.0%)	13 (6.0%)		
Job	Unemployed	110 (88.0%)	15 (12%)	2.455	0.117
	Employed	188 (93.1%)	14 (6.9%)		
Diabetes history	No	280 (90.6%)	29 (9.4%)	1.854	0.173
	Yes	18 (100.0%)	0 (0.0%)		
Thyroid history	No	248 (90.5%)	26 (9.5%)	0.794	0.373
	Yes	51 (94.4%)	3 (5.6%)		
Adverse pregnancy outcomes	No	259 (91.2%)	25 (8.8%)	0.013	0.909
	Yes	40 (90.9%)	4 (9.1%)		
Pregnancy Hyperlipidemia	No	252 (91.2%)	20 (8.8%)	3.567	0.059
	Yes	43 (82.7%)	9 (17.3%)		
Primipara	No	225 (93.0%)	17 (7.0%)	3.779	0.052
	Yes	74 (86.0%)	12 (14.0%)		
Hyperemesis Gravidarum	No	222 (94.5%)	13 (5.5%)	11.264	<0.001
	Yes	77 (82.8%)	16 (17.2%)		
Preterm birth	No	282 (92.5%)	23 (7.5%)	9.127	0.003
	Yes	17 (73.9%)	6 (26.1%)		
Casearean section	No	210 (93.8%)	14 (6.3%)	5.887	0.015
	Yes	89 (85.6%)	15 (14.4%)		
Gender	Female	173 (91.1%)	17 (8.9%)	0.052	0.819
	Male	102 (90.3%)	11 (9.7%)		
Fetal Macrosomia	No	276 (90.8%)	28 (9.2%)	0.551	0.458
	Yes	21(95.5%)	1(4.5%)		

### 3.2 Analysis of feature selection and prediction models

In the five imputed dataset, LASSO screened the most frequent variables (*n* > 3), including BMI, Edu, Job, DH, TH, PH, HG, PB, CS, Primipara as shown in Supplementary Table S2.

**Figure 3:**
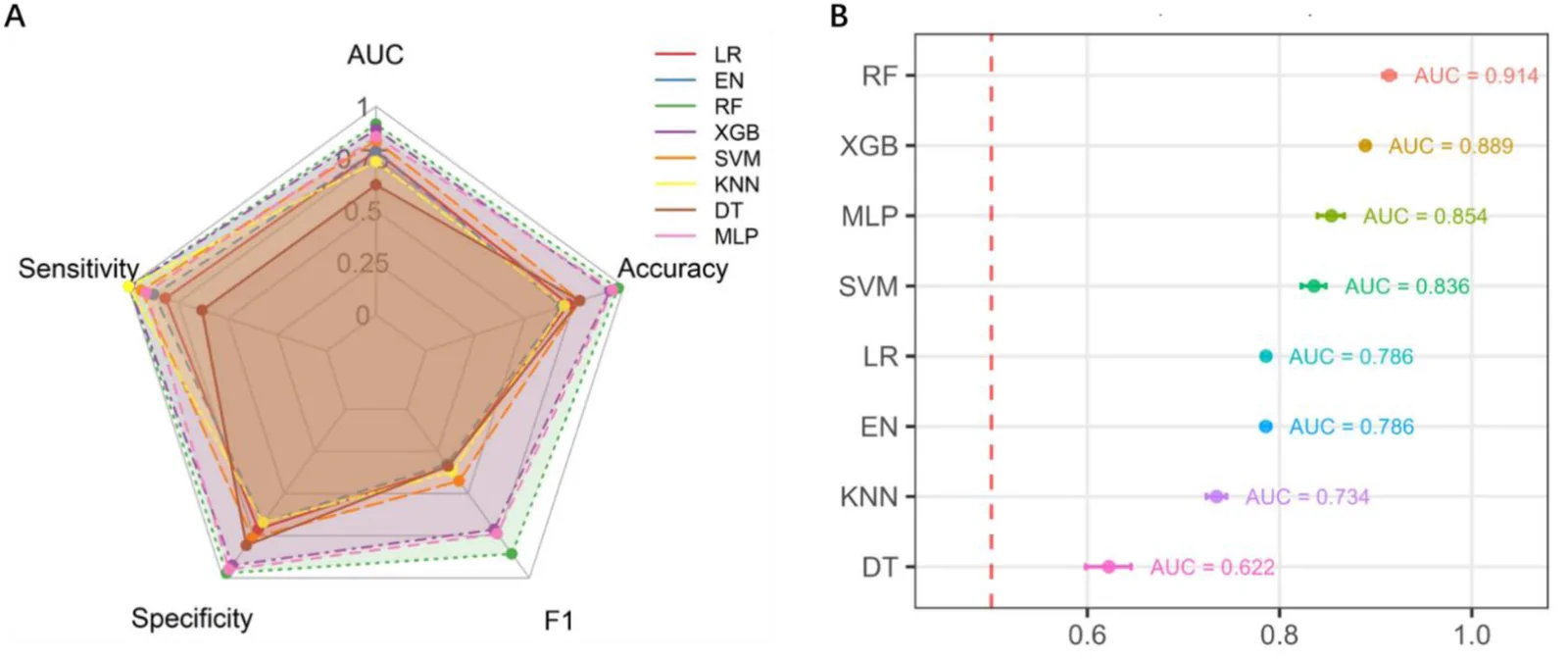
Performance evaluation results of each model in the interpolated dataset. **(A)** The radar chart of capacity assessment; **(B)** AUC by optimistic correction. DT, decision tree; EN, elastic net; KNN, k-nearest neighbor; LR, logistic regression; MLP, multilayer perceptron; RF, random forest; SVM, support vector machine; XGB, XGBoost.

**Figure 4:**
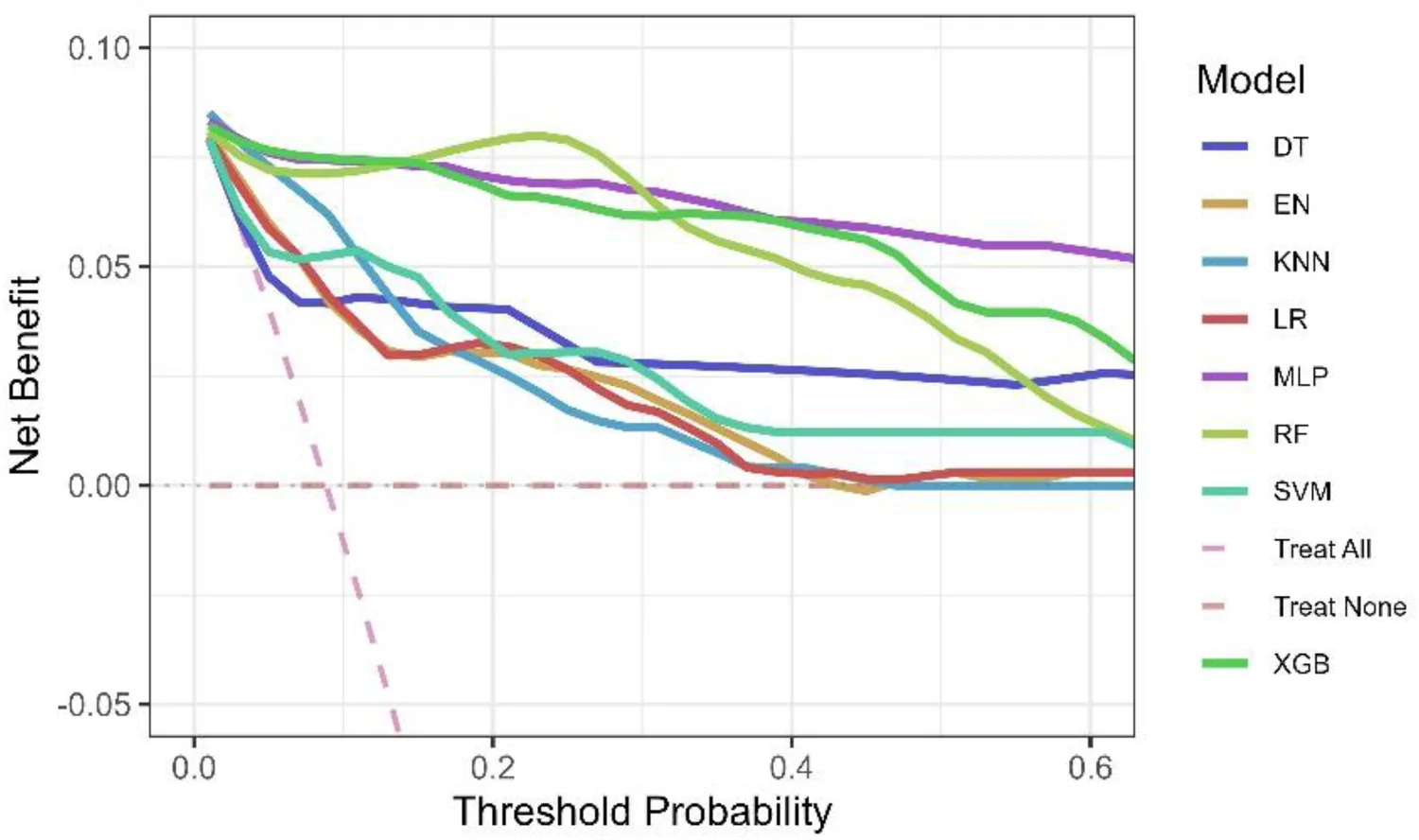
The decision curve analysis of the eight-prediction modes. DT, decision tree; EN, elastic net; KNN, k-nearest neighbor; LR, logistic regression; MLP, multilayer perceptron; RF, random forest; SVM, support vector machine; XGB, XGBoost.

Radar chart (Figure 3A) illustrated the sensitivity, specificity, F1, Accuracy, and optimistically corrected AUC values of eight predictive models on the imputed dataset, and the corresponding performance metrics were illustrated in Supplementary Table S3. The findings suggested that the RF model occupied the largest area, thereby indicating its superior predictive ability in comparison to other models, with the corrected AUC of 0.914 (Figure 3B). The XGB (AUC=0.889), SVM (AUC=0.836), and MLP (AUC=0.854) models demonstrated slightly diminished predictive capabilities, conversely, the DT exhibited the poorest performance (AUC = 0.622). DCA analysis revealed that all models are effective in predictions within the threshold range of 0–0.4, with RF, XGB, and MLP demonstrating relatively superior predictive abilities (Figure 4). However, none of the models were well calibrated as shown in Supplementary Figure S1.

### 3.3 Model performance

**Figure 5:**
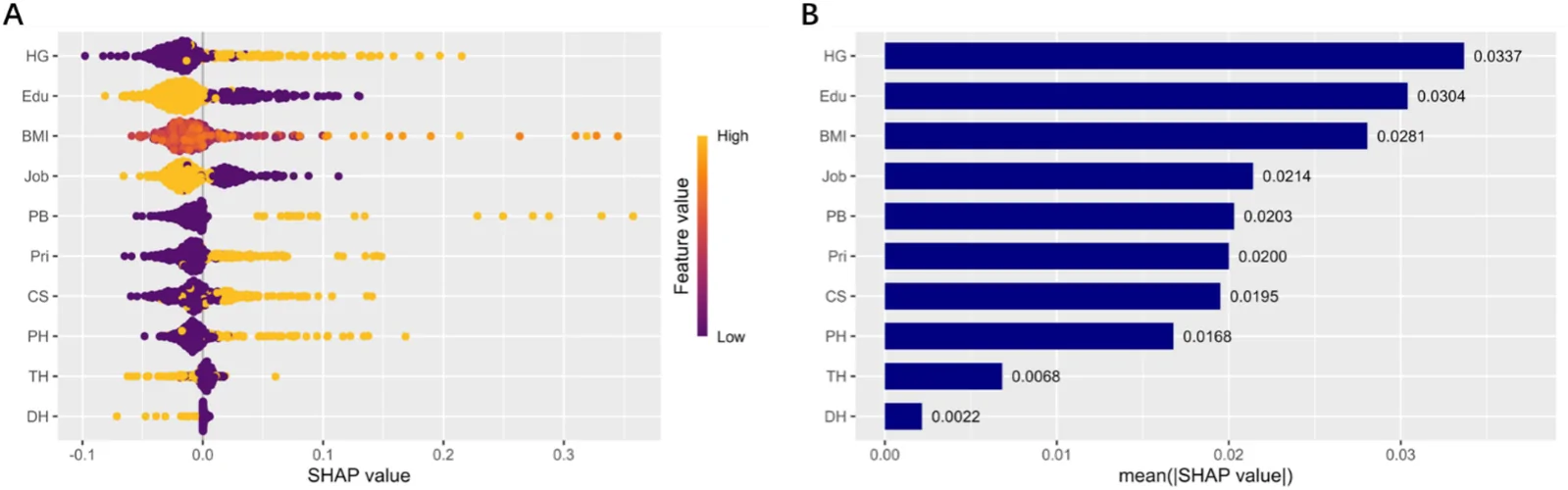
SHAP analysis results of random forest model. **(A)** Beeswarm results; **(B)** Feature importance results. APO, adverse pregnancy outcome; CS, caesarean section; DH, diabetes history; FM, fetal macrosomia; HG, hyperemesis gravidarum; NP, neonatal pneumonia; PB, preterm birth; PH, pregnancy hyperlipidemia; Pri, primipara; TH, thyroid abnormality history.

The SHAP analysis results of the RF model indicated that Edu, BMI, HG, Pri, PH, PB and job were important variables in predicting neonatal pneumonia (Figures 5A,B). In a particular instance, Edu, HG, Job, CS, PH and PB exerted a negative influence, while Pri, BMI, TH exerted a positive influence (Supplementary Figure S2).

**Figure 6:**
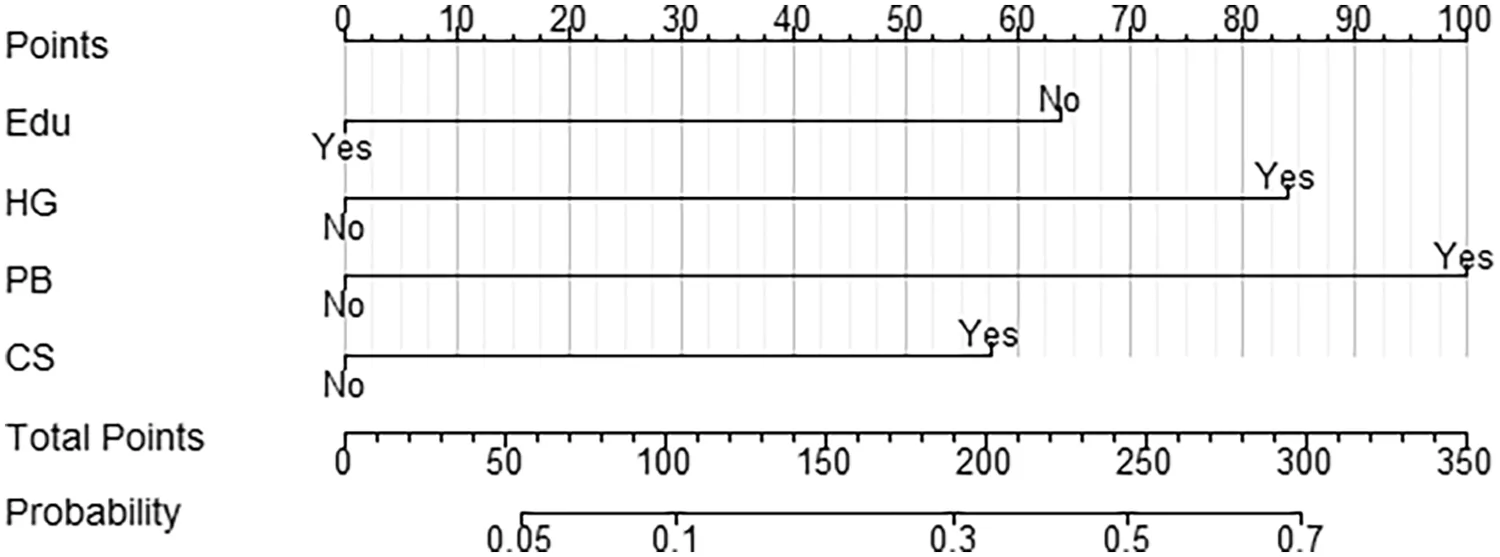
Presents an exploratory risk score constructed from the four variables retained in the logistic regression model. CS, caesarean section; HG, hyperemesis gravidarum.

Table 2 showed that, among the variables selected by LASSO, only Higher Edu (OR = 0.370, 95% *CI* = 0.158–0.865, *P* = 0.022), HG (OR = 3.706, 95% *CI* = 1.587–8.653, *P* = 0.002), PB (OR = 4.763, 95% *CI* = 1.522–14.901, *P* = 0.007), and Caesarean Section (CS) (OR = 2.456, 95% *CI* = 1.050–5.745, *P* = 0.038) were independent factors affecting neonatal pneumonia, *R*^2^= 0.189, Hosmer-Lemeshow Test *χ*^2^ = 7.099, *P* = 0.213. There appeared to be weak correlation between these surviving variables as shown in Supplementary Table S4. Figure 6 presented a simplified risk score model based on the four influencing factors. In the sensitivity analysis replacing dichotomous preterm birth with gestational age at delivery as a continuous predictor, higher gestational age was associated with lower odds of neonatal pneumonia (OR per 1-week increase = 0.700, 95% CI: 0.557–0.880, *P* = 0.002, Supplementary Table S5), which was directionally consistent with the increased risk observed for preterm birth in the primary analysis and supported the robustness of this association.

**Table 2:** Selected features influencing pneumonia risk were analyzed using logistic regression.

Variables	Crude OR (95% CI)	*P*	Adjusted OR (95% CI)	*P*	*VIF*
Age	0.982 (0.905, 1.066)	0.670			
BMI	1.083 (0.982, 1.193)	0.109			
Education (Higher)	0.370 (0.166, 0.824)	0.015	0.370 (0.158, 0.865)	0.022	1.012
Job (Employed)	0.668 (0.302, 1.478)	0.320			
Diabetes History (Yes)	0.000 (0.000, NA)	NA			
Adverse Pregnancy (Yes)	1.163 (0.380, 3.562)	0.792			
Primipara (Yes)	2.115 (0.935, 4.784)	0.072			
Thyroid disorder History (Yes)	0.576 (0.167, 1.989)	0.383			
Pregnancy Hyperlipidemia (Yes)	2.341 (0.961, 5.704)	0.061			
Hyperemesis Gravidarum (Yes)	3.845 (1.713, 8.628)	0.001	3.706 (1.587, 8.653)	0.002	1.010
Preterm (Yes)	4.518 (1.599, 12.763)	0.004	4.763 (1.522, 14.901)	0.007	1.008
Caesarean Section (Yes)	2.500 (1.125, 5.555)	0.025	2.456 (1.050, 5.745)	0.038	1.013
Gender (Boy)	1.200 (0.535, 2.688)	0.658			
Macrosomia (Yes)	0.506 (0.065, 3.935)	0.515			

## 4 Discussion

In study of 328 mother–infant pairs with GDM, we developed and internally validated eight machine learning-based prediction models for neonatal pneumonia using variables routinely available in primary care. The Random Forest model achieved the highest corrected AUC (0.914), while logistic regression identified preterm birth, hyperemesis gravidarum, cesarean section, and higher maternal education level as independent factors. Although the model's predictive performance is promising, its clinical utility lies not in definitive diagnosis but in enabling risk-stratified monitoring—a function particularly valuable in resource-limited settings where advanced diagnostics are unavailable.

### 4.1 Mother's education level

The available evidence does not support maternal education level as a direct biological determinant of neonatal pneumonia. Rather, in pregnancies complicated by GDM, educational attainment may serve as a contextual marker of broader social and healthcare-related factors, including health literacy, access to reliable health information, availability of diabetes education, continuity of antenatal care, and opportunities for self-management support (30). These factors are particularly relevant in GDM, for which effective management involves dietary modification, glucose monitoring, medication when indicated, and regular antenatal follow-up. Recent GDM-specific studies have identified health literacy as an important correlate of self-management, and maternal educational attainment has been associated with differences in health literacy and glycemic control among women with GDM (31, 32). This relationship may also be relevant to neonatal infection susceptibility because maternal GDM has been associated with an increased risk of infection in offspring, suggesting that exposure to the intrauterine diabetic environment may influence early immune protection (14). Accordingly, the association observed in our study may reflect differences in access to and support for effective GDM management rather than an effect of education itself. In primary healthcare settings, maternal education level may therefore be more appropriately interpreted as a marker of potential unmet informational, healthcare, or social support needs. This finding supports the provision of clear, accessible, and health-literacy-sensitive GDM counselling, including the use of plain language, visual materials, and reinforced postnatal education regarding neonatal respiratory warning signs, according to individual needs. Importantly, health literacy, healthcare access, socioeconomic circumstances, and longitudinal glycemic control were not directly measured in our study. Therefore, these pathways remain plausible explanations rather than demonstrated causal mechanisms.

### 4.2 Hyperemesis gravidarum

HG may be associated with insufficient nutrient intake, electrolyte imbalance, and weight loss, which may adversely affect fetal alveolarization and angiogenesis, associated with reduced lung function reserve and weakened respiratory defences in newborns. Experimental evidence supports this plausibility: vitamin A deficiency induces pulmonary inflammation and impairs viral clearance (33), while maternal vitamin D deficiency may cause persistent structural lung damage in offspring (34). These mechanisms provide a biological basis for the significant association between HG and neonatal pneumonia observed in our study and by others (35). However, the novelty of our finding lies not in confirming this association, but in quantifying HG's contribution within a composite risk score specifically for GDM pregnancies—a population already vulnerable to immune dysregulation via sustained HIF-1*α* activation and reduced IgG antibody diversity (14). In GDM complicated by HG, both maternal metabolic immune dysfunction and nutritional deficiency may jointly contribute to heightened neonatal pneumonia susceptibility. Clinically, the value of identifying HG as a risk factor lies not in prompting interventions already considered standard care (e.g., hydration and antiemetic therapy), but in risk stratification—prioritizing neonates born to mothers with GDM and HG for enhanced postnatal respiratory monitoring and targeted parental education. We acknowledge, however, a critical limitation: our model captured HG only as a binary variable (presence vs. absence), without accounting for severity grading (e.g., PUQE score), treatment intensity (antiemetic use, hospitalization), weight loss percentage, ketonuria duration, or vitamin supplementation status. These missing granularities likely introduce misclassification and may obscure dose-response relationships. Future prospective studies should incorporate validated severity metrics and nutritional biomarkers (e.g., serum vitamin A/D levels) to refine predictive accuracy. Until then, we recommend that clinicians interpret HG as a “risk signal” warranting heightened awareness rather than a deterministic predictor, consistent with the preliminary, hypothesis-generating nature of our findings.

### 4.3 Preterm birth

Preterm birth is a well-established risk factor for neonatal respiratory infection because incomplete pulmonary development, reduced respiratory reserve, and immaturity of both innate and adaptive immunity can compromise host defense against respiratory pathogens (36). When respiratory support is required, exposure to invasive respiratory devices may further increase the risk of pulmonary infection (37). In the context of GDM, however, prematurity may represent more than an isolated gestational-age effect. Recent evidence suggests that maternal diabetes is associated with altered early-life immune protection and an increased risk of infection in offspring; notably, the diabetes-associated increase in infection susceptibility was particularly evident among infants born preterm (14). Thus, preterm neonates born to mothers with GDM face concurrent risk factors, including developmental immaturity of the lungs and immune system alongside metabolic perturbations associated with the intrauterine hyperglycemic environment (38). This framework may help explain the strong association between preterm birth and neonatal pneumonia observed in our GDM cohort (adjusted OR = 4.763). Clinically, preterm neonates born to mothers with GDM may therefore constitute a particularly vulnerable subgroup warranting close postnatal respiratory and infection surveillance. However, because all mothers in our cohort had GDM and longitudinal measures of maternal glycemic control were unavailable, our study cannot determine whether GDM statistically modifies the effect of prematurity. The proposed amplification should therefore be regarded as a biologically plausible, hypothesis-generating interpretation rather than a demonstrated interaction.

### 4.4 Cesarean section

Cesarean section deprives newborns of the opportunity to come into contact with the mother's vaginal and intestinal microbiota during delivery and may lead to delayed and disrupted colonization of the newborn's intestinal and respiratory microbiota (39, 40). Consequently, cesarean delivery may contribute to neonatal respiratory vulnerability which may be complicated by GDM. Cesarean delivery performed before the onset of labor avoids the physiological and hormonal adaptations of labor that promote neonatal lung-fluid absorption, potentially increasing early respiratory morbidity and the need for respiratory support. Importantly, this relationship has been demonstrated specifically in a cohort of 581 neonates born at ≥34 weeks to mothers with GDM, exposure to labor was associated with a lower frequency of respiratory morbidity requiring NICU admission than delivery without labor (4.9% vs. 10.4%; adjusted OR = 0.41) (41). In addition, both delivery mode and maternal GDM can influence the establishment of the neonatal microbiota (42), while recent translational evidence indicates that GDM-associated neonatal dysbiosis is accompanied by altered inflammatory responses and increased susceptibility to infection (43). Consistent with this combined-risk concept, the association between maternal diabetes and early-life infection was more pronounced among infants delivered by cesarean section (14). Collectively, these observations indicate that cesarean-related impairment of respiratory transition and altered microbial colonization may further compound the baseline risk established by maternal GDM, potentially increasing overall susceptibility to neonatal pneumonia. Nevertheless, our study did not distinguish elective from intrapartum cesarean delivery and lacked information on intrapartum antibiotic exposure. Therefore, cesarean section should be interpreted as a risk marker rather than as a reason to alter medically indicated delivery decisions, and unnecessary cesarean delivery should be avoided according to standard obstetric indications.

### 4.5 Model performance

The eight machine learning models in this study demonstrate equivalent performance in the context of the clinical background of low incidence of neonatal pneumonia (8.8%). Firstly, the conventional single classifier model is susceptible to the production of prediction bias that is biased towards the majority when processing imbalanced medical data. Conversely, the bagging-based random forest can effectively reduce the prediction variance of minority samples through self-help sampling and a majority voting mechanism. In circumstances where the EPV (approximately 3) is far below 10–20, the RF significantly reduces the risk of overfitting by building multiple decision trees and aggregating their prediction results (44), while the DT is prone to ignoring minority characteristics due to a lack of integration mechanism, and may be associated with a near random guess level (45). The XGB algorithm is predicated on the gradual optimisation of the weighting of erroneous classification samples. In scenarios where the number of positive samples is limited, the model is susceptible to local optimisation, owing to the absence of sufficient “wrong” samples to facilitate iterative optimisation (46). Moreover, recent advances in GDM risk prediction, such as the GDMPredictor developed have demonstrated the utility of machine learning approaches for early identification of at-risk pregnancies (47). Our study may complement these efforts by focusing on neonatal outcomes rather than maternal GDM diagnosis, and by prioritizing variables readily available in primary care settings.

### 4.5 Clinical utility of the risk stratification approach

The potential value of a future simplified risk-stratification tool informed by these findings would lie not in replacing existing clinical standards, but in helping primary care physicians prioritize monitoring intensity for neonates born to GDM mothers. Ideally, this risk score could be calculated upon admission to the postpartum ward using information already documented in the medical record, allowing nursing staff to identify high-risk neonates for enhanced respiratory monitoring without requiring additional laboratory tests. In low-resource settings, where laboratory and imaging resources are constrained, risk stratification based on readily ascertainable maternal factors can help optimize limited healthcare resources. This aligns with the World Health Organization's Integrated Management of Childhood Illness (IMCI) framework (48), which employs simple clinical algorithms to identify infants requiring further evaluation at the primary care level. Recent studies have demonstrated that machine learning models can effectively predict adverse neonatal outcomes in GDM pregnancies (49), for example, Houri et al. reported prediction accuracies of 82% at GDM diagnosis and 91% at delivery using a neural network algorithm, with composite outcomes including respiratory distress syndrome and NICU admission (50). Similarly, risk scores developed for neonatal mortality prediction have shown clinical utility through decision curve analysis, supporting their use in guiding timely interventions (51).

However, unlike diagnostic algorithms that directly trigger therapeutic decisions, our risk score is intended to inform surveillance intensity rather than specific interventions. For a neonate identified as high-risk, recommended actions would include more frequent respiratory status assessments during the first 72 h; structured parental education on pneumonia danger signs; and a lower threshold for chest ultrasound or referral (17). We emphasize that this framework remains preliminary: the risk factors we identified—preterm birth, HG, cesarean section, and lower education—are already associated with multiple adverse neonatal outcomes beyond pneumonia. Consequently, the specificity of this risk score to neonatal pneumonia, as opposed to general neonatal morbidity, remains unproven and requires external validation in larger multicentre cohorts before any clinical application can be considered.

### 4.6 Limitation

The most critical limitation of this study is the small sample size, particularly the insufficient number of positive events (*n* = 29). For the 9-predictor model, the events-per-variable (EPV) ratio was approximately 3, well below the conventional minimum of 10 and far below the EPV ≥ 20 recommended for machine learning models (22, 52). Future work must prioritize multi-center data collection to achieve adequate statistical power. Secondly, due to the objective limitation of relatively lagging informationization construction in primary healthcare institutions (53), some key clinical variables with important predictive value are severely missing, such as maternal blood glucose control level during pregnancy (such as glycated hemoglobin) (54), inflammation related biomarkers (such as C-reactive protein and procalcitonin) (55), neonatal immune indicators (such as umbilical cord IgG antibody spectrum, IL, 17A levels), and antibiotic use during delivery. The absence of these variables may compromise the model's predictive efficacy. Thirdly, although this study adhered to the TRIPOD guidelines and compared multiple machine learning algorithms (56), internal validation frequently overestimates the model's genuine predictive capacity. Furthermore, the model's performance in other primary healthcare institutions, across diverse geographical regions, and among varied populations at different time points remains to be elucidated. This undermines the model's reliability for clinical promotion. Fourthly, although the SHAP method was employed in this study to enhance model interpretability, the level of informatisation in primary healthcare institutions and the acceptance of machine learning models among medical personnel still require further evaluation. While certain variables incorporated into the model (e.g., preterm, severe vomiting during pregnancy, and education level) show statistical associations with neonatal pneumonia, the underlying causal mechanisms remain to be fully elucidated. This is particularly salient given the potential confounding influence of education level, a socio-economic composite indicator, which may be intertwined with factors such as medical accessibility and health literacy in its association with pneumonia. Consequently, a cautious approach should be adopted when interpreting clinical interventions. In addition, because our cohort consisted exclusively of pregnancies complicated by GDM and lacked longitudinal indices of maternal glycemic control, the proposed amplification of education-, prematurity-, and delivery-related risks by the GDM environment should be interpreted as mechanistically plausible rather than as statistically demonstrated effect modification. In summary, the predictive model constructed in this study still needs to be externally validated in a prospective cohort of multiple centres and large samples before being applied in clinical practice. Furthermore, our model should therefore be interpreted as an exploratory subgroup-specific prediction model rather than a substitute for universal neonatal pneumonia prediction models. Future studies comparing GDM and non-GDM pregnancies are required to determine whether GDM modifies the relative contribution of established neonatal pneumonia risk factors.

## 5 Conclusion

This study provides preliminary evidence that routinely available maternal and perinatal clinical variables may support the future development of a simplified risk-stratification tool for neonatal pneumonia among pregnancies complicated by GDM in primary healthcare settings. Preterm birth, hyperemesis gravidarum, cesarean section, and maternal educational attainment emerged as candidate predictors in the present cohort; however, they should not be regarded as a definitive predictor set. Among the eight machine learning models evaluated, Random Forest demonstrated the best discrimination in internal validation, although the limited sample size, small number of pneumonia events, and single-center design preclude immediate clinical application. Larger multicentre prospective studies, ideally including external populations and non-GDM comparison groups, are needed to refine predictor selection, evaluate model stability and calibration, and externally validate the resulting risk-stratification approach before routine clinical use.

## Data Availability

The original contributions presented in the study are included in the article/Supplementary Material, further inquiries can be directed to the corresponding author. The code for this study has been hosted on the GitHub opensource platform (https://github.com/Tomcp85/Pneumonia- Prediction.git).
