# Supplementary material for: Developing a prediction model of neonatal pneumonia in patients with gestational diabetes mellitus in primary medical institutions
Source: Front Pediatr. 2026 Aug 25;14:1840176. doi: 10.3389/fped.2026.1840176 (PMC13550188; doi:10.3389/fped.2026.1840176)
Supplement: Supplementary file 1 [file Supplementaryfile1.docx]

**Figure S1. The calibration of each model.** DT: Decision Tree; EN: elasticNet; KNN: K-Nearest Neighbor; LR: Logistic Regression; MLP: Multi-Layer Perceptron; RF: Random Forest; SVM: Support Vector Machine; XGB: XGBoost.

**Figure S2. Example of Waterfall Chart in SHAP Analysis.** CS: caesarean section; DH: diabetes history; FM: fetal macrosomia; HG: hyperemesis gravidarum; NP: neonatal pneumonia; PB: preterm birth; PH: pregnancy hyperlipidemia; Pri: primipara; TH: thyroid abnormalities history.

| **Table S1. Comparison of original and imputed datasets for assessment of missing data handling.** | | | | | | |  |
| --- | --- | --- | --- | --- | --- | --- | --- |
|  |  | **Raw dataset** | | **Imputed dataset** |  |  | |
|  |  | (n=328) | Missing | (n=328) | ***t* or *χ^2^*** | **P** | |
| **Mother** |  |  |  |  |  |  | |
| Age (years) |  | 31.22±4.93 | 0 | 31.22±4.93 | 0 | 1.000 | |
| BMI (kg/m^2^) |  | 24.43±3.79 | 0 | 24.43±3.79 | 0 | 1.000 | |
| Education Level | Lower | 110 | 3(0.9%) | 112 | 0.007 | 0.935 | |
|  | Higher | 215 |  | 216 |  |  | |
| Job | Unemployed | 125 | 1(0.3%) | 125 | 0.001 | 0.976 | |
|  | Employed | 202 |  | 202 |  |  | |
| Disease History |  |  |  |  |  |  | |
| Diabetes | No | 309 | 1(0.3%) | 310 | <0.001 | 0.992 | |
|  | Yes | 18 |  | 18 |  |  | |
| Thyroid Disorder | No | 274 | 0 | 274 | 0 | 1.000 | |
|  | Yes | 54 |  | 54 |  |  | |
| Adverse Pregnancy | No | 284 | 2(0.6%) | 285 | 0.007 | 0.931 | |
|  | Yes | 42 |  | 43 |  |  | |
| Present Pregnancy |  |  |  |  |  |  | |
| Hyperlipidemia | No | 272 | 4(1.2%) | 274 | 0.021 | 0.886 | |
|  | Yes | 52 |  | 54 |  |  | |
| Primipara | No | 242 | 0 | 242 | 0 | 1.000 | |
|  | Yes | 86 |  | 86 |  |  | |
| Hyperemesis Gravidarum | No | 235 | 0 | 235 | 0 | 1.000 | |
|  | Yes | 93 |  | 93 |  |  | |
| Caesarean Section | No | 224 | 0 | 224 | 0 | 1.000 | |
|  | Yes | 104 |  | 104 |  |  | |
| **Infants** |  |  |  |  |  |  | |
| Preterm | No | 305 | 0 | 305 | 0 | 1.000 | |
|  | Yes | 23 |  | 23 |  |  | |
| Gender | Female | 190 | 25(7.6%) | 203 | 0.059 | 0.808 | |
|  | Male | 113 |  | 125 |  |  | |
| Macrosomia | No | 304 | 2(0.6%) | 305 | 0.018 | 0.894 | |
|  | Yes | 22 |  | 23 |  |  | |
| Pneumonia | No | 299 | 0 | 299 | 0 | 1.000 | |
|  | Yes | 29 |  | 29 |  |  | |

All 328 mother-infant pairs meeting the inclusion criteria were retained in the analysis. Missing values were handled using multiple imputation with predictive mean matching, generating five complete datasets. This table presents the original data with missing values (Raw dataset, n=328) alongside the first imputed dataset (Imputed dataset, n=328) as an illustrative example. No significant differences were observed between raw and imputed data (all P > 0.7), supporting the validity of the imputation procedure.

**Table S2. The selected variables in each imputation procedure.**

|  | **Imputed sequence** | | | | |  |
| --- | --- | --- | --- | --- | --- | --- |
| **Variables** | **1** | **2** | **3** | **4** | **5** | **Total** |
| Age | 0 | 0 | 0 | 0 | 0 | 0 |
| BMI | 1 | 1 | 1 | 1 | 1 | 5 |
| Education | 1 | 1 | 1 | 1 | 1 | 5 |
| Job | 1 | 1 | 1 | 1 | 1 | 5 |
| Diabetes History | 1 | 1 | 1 | 1 | 1 | 5 |
| Thyroid Abnormalities | 1 | 1 | 1 | 1 | 1 | 5 |
| Pregnancy Hyperlipidemia | 1 | 1 | 1 | 1 | 1 | 5 |
| Hyperemesis Gravidarum | 1 | 1 | 1 | 1 | 1 | 5 |
| Primipara | 1 | 1 | 1 | 1 | 1 | 5 |
| Caesarean Section | 1 | 1 | 1 | 1 | 1 | 5 |
| Preterm Birth | 1 | 1 | 1 | 1 | 1 | 5 |
| Adverse Pregnancy Outcome | 0 | 0 | 0 | 0 | 0 | 0 |
| Sex | 0 | 0 | 0 | 0 | 0 | 0 |
| Fetal Macrosomia | 1 | 0 | 1 | 1 | 0 | 3 |
| **Total** | 11 | 10 | 11 | 11 | 10 | **53** |

**Table S3. Average performance of each model on imputed datasets by bootstrap validation (n=200).**

| **Model** | **Sensitivity** | **Specificity** | **F1** | **Accuracy** | **Youden’s J** | **Corrected AUC** |
| --- | --- | --- | --- | --- | --- | --- |
| **DT** | 0.629 (0.552, 0.700) | 0.808 (0.525, 0.941) | 0.338 (0.215, 0.488) | 0.777 (0.588, 0.895) | 0.398 (0.342, 0.458) | 0.622 (0.598, 0.645) |
| **EN** | 0.872 (0.720, 0.948) | 0.671 (0.582, 0.750) | 0.327 (0.296, 0.359) | 0.687 (0.613, 0.754) | 0.524 (0.496, 0.552) | 0.786 (0.781, 0.790) |
| **KNN** | 0.999 (0.999, 0.999) | 0.672 (0.666, 0.677) | 0.371 (0.368, 0.375) | 0.701 (0.696, 0.706) | 0.672 (0.666, 0.677) | 0.734 (0.724, 0.745) |
| **LR** | 0.814 (0.790, 0.836) | 0.713 (0.694, 0.731) | 0.341 (0.327, 0.354) | 0.721 (0.705, 0.738) | 0.526 (0.501, 0.551) | 0.786 (0.782, 0.790) |
| **MLP** | 0.909 (0.808, 0.959) | 0.951 (0.883, 0.980) | 0.739 (0.601, 0.841) | 0.943 (0.888, 0.972) | 0.839 (0.785, 0.881) | 0.854 (0.839, 0.867) |
| **RF** | 0.996 (0.955, 1.000) | 0.971 (0.921, 0.990) | 0.858 (0.723, 0.933) | 0.971 (0.933, 0.988) | 0.950 (0.945, 0.955) | 0.914 (0.907, 0.920) |
| **SVM** | 0.935 (0.894, 0.960) | 0.763 (0.754, 0.772) | 0.426 (0.410, 0.442) | 0.778 (0.768, 0.787) | 0.695 (0.659, 0.729) | 0.836 (0.823, 0.848) |
| **XGB** | 0.996 (0.955, 1.000) | 0.925 (0.886, 0.951) | 0.713 (0.634, 0.782) | 0.930 (0.898, 0.952) | 0.909 (0.891, 0.923) | 0.889 (0.885, 0.894) |

| **Table S4. The correlation matrix of variables in the final binary logistic regression model.** | | | | | |
| --- | --- | --- | --- | --- | --- |
|  | Constant | Education (Higher) | Hyperemesis Gravidarum (Yes) | Preterm (Yes) | Caesarean Section (Yes) |
| Constant | 1 | -0.422 | -0.504 | -0.283 | -0.432 |
| Education (Higher) | -0.422 | 1 | -0.029 | 0.002 | -0.104 |
| Hyperemesis Gravidarum (Yes) | -0.504 | -0.029 | 1 | 0.086 | -0.031 |
| Preterm (Yes) | -0.283 | 0.002 | 0.086 | 1 | 0.027 |
| Caesarean Section (Yes) | -0.432 | -0.104 | -0.031 | 0.027 | 1 |

**Table S5. The sensitivity analysis of the binary logistic model with delivery week instead of preterm birth.**

|  | ***B*** | ***S.E.*** | ***Wald χ^2^*** | **d*f*** | ***P*** | ***OR*** | ***OR_Lower_*** | ***OR_Uppper_*** | ***VIF*** |
| --- | --- | --- | --- | --- | --- | --- | --- | --- | --- |
| Edu (Yes) | -1.038 | 0.44 | 5.561 | 1 | 0.018 | 0.354 | 0.149 | 0.839 | 1.006 |
| HG (Yes) | 1.333 | 0.438 | 9.253 | 1 | 0.002 | 3.793 | 1.607 | 8.953 | 1.012 |
| CS (Yes) | 0.939 | 0.439 | 4.565 | 1 | 0.033 | 2.557 | 1.081 | 6.050 | 1.012 |
| Delivery Week | -0.356 | 0.117 | 9.303 | 1 | 0.002 | 0.700 | 0.557 | 0.880 | 1.011 |

Hosmer and Lemeshow Test *χ^2^* = 7.422, *P* = 0.492; Nagelkerke *R*^2^ = 0.213.
